# Acoustic Radiation Force Impulse Technology in the Differential Diagnosis of Solid Breast Masses with Different Sizes: Which Features Are Most Efficient?

**DOI:** 10.1155/2015/410560

**Published:** 2015-07-16

**Authors:** Min Bai, Hui-Ping Zhang, Jin-Fang Xing, Qiu-Sheng Shi, Ji-Ying Gu, Fan Li, Hui-Li Chen, Xue-Mei Zhang, Yun Fang, Lian-Fang Du

**Affiliations:** ^1^Department of Ultrasound, Shanghai First People's Hospital, Shanghai Jiao Tong University, Shanghai 200080, China; ^2^Department of Pathology, Shanghai First People's Hospital, Shanghai Jiao Tong University, Shanghai 200080, China; ^3^School of Mathematics and Science, Shanghai Normal University, Shanghai 200234, China

## Abstract

*Purpose*. To evaluate diagnostic performance of acoustic radiation force impulse (ARFI) technology for solid breast masses with different sizes and determine which features are most efficient. *Materials and Methods*. 271 solid breast masses in 242 women were examined with ARFI, and their shear wave velocities (SWVs), Virtual Touch tissue imaging (VTI) patterns, and area ratios (ARs) were measured and compared with their histopathological outcomes. Receiver operating characteristic curves (ROC) were calculated to assess diagnostic performance of ARFI for small masses (6–14 mm) and big masses (15–30 mm). *Results*. SWV of mass was shown to be positively associated with mass size (*P* < 0.001). For small masses, area under ROC (Az) of AR was larger than that of SWV (*P* < 0.001) and VTI pattern (*P* < 0.001); no significant difference was found between Az of SWV and that of VTI pattern (*P* = 0.906). For big masses, Az of VTI pattern was less than that of SWV (*P* = 0.008) and AR (*P* = 0.002); no significant difference was identified between Az of SWV and that of AR (*P* = 0.584). *Conclusions*. For big masses, SWV and AR are both efficient measures; nevertheless, for small masses, AR seems to be the best feature.

## 1. Introduction

Breast cancer is common; its incidence has kept rising in China in the recent years and has now, for instance, occupied the first place in the list of malignant tumors occurring to women in Shanghai [1]. However, Chinese women are characterized by having dense breast tissue that X-ray cannot easily penetrate. Hence, ultrasound (US) plays an important role in the diagnosis of breast cancer by Chinese practitioners [2].

Acoustic radiation force impulse (ARFI) imaging is a new ultrasonic elasticity technology. Compared with static elastography, ARFI can yield highly reproducible information on the tissue stiffness of solid breast masses within and across observers [3–5]. It incorporates two different modes: Virtual Touch tissue quantification (VTQ) and Virtual Touch tissue imaging (VTI) [6]. VTQ is quantitative, which tracks a shear wave in the region of interest (ROI) that travels perpendicular to the direction of the acoustic push pulse and calculates the shear wave velocity (SWV, measured in m/s). The stiffer the tissue is, the greater the SWV will be [6]. VTI is qualitative, employing a short acoustic impulse of high intensity to deform the tissue elements in a defined ROI and creating a static map (i.e., elastogram) in grayscale of the relative stiffness of the tissues. The stiffer the tissue is, the darker the mass area becomes [6]. VTI images can be classified as different patterns and calculated ratios of VTI-to-B-mode mass areas (area ratio, AR). Several studies have demonstrated that SWV, VTI pattern, and AR are useful in differentiating between benign and malignant solid breast masses [4–9]. However, no studies have compared the diagnostic performance of these features.

In a number of previous studies, the association between stiffness and tumor size was recorded. With strain elastography, Giuseppetti et al. noticed that the histotypes and sizes of the lesions have a strong influence on their degree of stiffness [10]. Evans et al., with shear wave elastography, found that average mean stiffness for malignancies less than 15 mm in diameter was 109 kPa, compared with 167 kPa in cancers larger than 15 mm [11]. However, to our knowledge, no prior studies have assessed the value of ARFI in the differential diagnosis of breast masses with different tumor sizes. In our current study, we have adopted ARFI imaging and introduced SWV, VTI pattern, and AR, aiming to evaluate their diagnostic performance among different size ranges of solid breast masses (e.g., 6–14 mm and 15–30 mm) and determine which features are most efficient.

## 2. Materials and Methods

### 2.1. Patients

The prospective study was approved by the Ethics Committee of our hospital and all participants provided written informed consent. From June 2013 to June 2014, a total of 318 women were enrolled in the study consecutively. All of the patients were recruited on the basis that they had been suspected to have solid breast masses based on conventional US examinations. 468 masses were evaluated and recorded. The following exclusion criteria were applied: (1) masses with maximum diameters smaller than 6 mm, because of the VTQ ROI having a fixed dimension of 5 mm × 6 mm; (2) masses with maximum diameters larger than 30 mm, because the maximum width of the VTI ROI is 38 mm; (3) masses from patients having received chemotherapy or radiotherapy treatment before; (4) masses which were not resected by surgery; (5) masses proven to be not solid after resection. Hence, a total of 242 women (age range: 17–85 years; mean age: 46.4 years) with 271 solid breast masses finally constituted the study group. According to the maximum diameters less than 15 mm or not, the masses were divided into a small-size group and a large-size group. 111 masses were classified into the small-size group and 160 masses into the large-size group.

### 2.2. Conventional US and ARFI Elastography

All US examinations were performed by three radiologists (14, 11, and 14 years of experience in breast US, resp., and 3-year experience with ARFI elastography) independently; the VTQ and the VTI of a mass were done by the same radiologist. Both the conventional US and ARFI assessments were performed with a Siemens ACUSON S2000 ultrasound system (Siemens Medical Solutions, Mountain View, CA, USA) using a 9 L4 linear transducer (4–9 MHz).

During the study, conventional US was performed to scan the patient's breast thoroughly prior to ARFI assessment. With the relevant arm raised and the patient taking supine or lateral position, so as to flatten the breast on the chest wall, the maximum diameters of individual masses were measured. The planes of maximum diameters were selected for ARFI assessment.

For ARFI assessment, the transducer was gently placed onto the skin surface with no pressure. After activating VTQ or VTI, the transducer was kept still and the patient was asked to hold her breath during the acquisition of SWVs and VTI images.

In the VTQ mode, ROIs were localized to the selected plane to evaluate an average stiffness of the whole mass. Each ROI was placed within the mass and avoids thick calcifications as much as possible. Nine valid SWV values were obtained from an individual mass, and the median value was recorded as a mean SWV. Data collected were evaluated by another radiologist (6-year experience in breast US and 2-year experience with ARFI elastography) blinded to clinical information, other US, and pathology results.

In the VTI mode, the ROI was set to include the mass and its adequate surrounding normal breast tissue. A VTI image was displayed side by side with a corresponding B-mode image, and the boundaries of the mass in both images may not overlap because of the different tissue contrast mechanisms employed [4, 8]. In the present study, VTI images were obtained three times at each site. All VTI images were evaluated by another radiologist (13-year experience in breast US and 2-year experience with ARFI elastography) who was blind to clinical information, other US, and pathology results.

**Figure 1:**
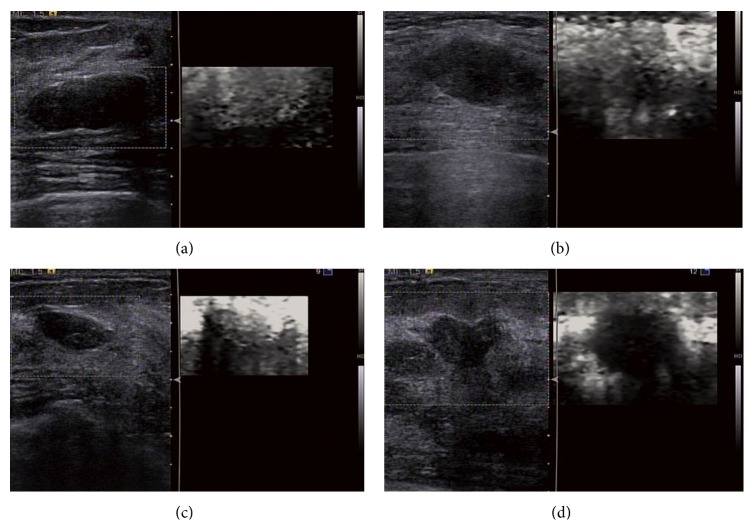
Images of the VTI Patterns 1–4. (a) VTI (right) shows a bright area (Pattern 1) corresponding to the hypoechoic mass in B-mode US (left); (b) VTI (right) shows no bright or dark areas (Pattern 2) corresponding to the hypoechoic mass in B-mode US (left); (c) VTI (right) shows a dark (grey) area (Pattern 3) corresponding to the hypoechoic mass in B-mode US (left); (d) VTI (right) shows a dark (black) area (Pattern 4) corresponding to the hypoechoic mass in B-mode US (left).

Masses that failed to be visually confirmed on VTI images were classified as Pattern 2. Among the masses that were visually confirmed, those that were brighter than the surrounding breast tissue were classified as Pattern 1 and those darker than the surrounding breast tissue as Patterns 3 and 4. For those dark masses, differentiation was made between the gray ones (i.e., Pattern 3) and the black ones (i.e., Pattern 4) (Figure 1). For Patterns 1, 3, and 4, the areas of a mass in the VTI image and the B-mode image were both measured to calculate an AR.

### 2.3. Statistical Analysis

Data were expressed as mean ± SD. The chi-square test was used to analyze group differences of VTI pattern. The SWVs and ARs of the masses of multiple subgroups were compared using Kruskal-Wallis test, and any two groups were compared with Mann-Whitney *U* test. As multiple pairwise tests were performed for a single set of data, a Bonferroni correction was used to reduce the risk of false-positive results. A Bonferroni-adjusted significance level of 0.0083 was calculated to compare SWVs and ARs of the mass of multiple subgroups (6 hypotheses tested). The correlation between SWV and mass size was assessed by Bivariate-Correlations analysis.

The receiver operating characteristic curves (ROC) for SWVs, VTI patterns, and ARs of masses were plotted, and the optimal cutoff points were obtained by maximizing Youden's index which equals “sensitivity + specificity − 1.” The diagnostic performance of the selected criteria was evaluated in terms of sensitivity, specificity, positive predictive value (PPV), negative predictive value (NPV), accuracy, and the area under the receiver operating characteristic curve (AUROC, Az) with the 95% confidence interval (CI).

Finally, the *z* test was used to compare the Azs of SWV, VTI pattern, and AR. Az was compared by using a paired design and excluding patients with missing data. Missing data were present for AR, because ARs were unavailable for masses of Pattern 2. A Bonferroni correction was also used and an adjusted significance level of 0.0167 was calculated (3 hypotheses tested).

All analyses were performed with SPSS (version 11.0) for Windows (SPSS Inc., Chicago, IL, USA). Except for multiple pairwise comparison of SWVs (*P* < 0.0083), ARs (*P* < 0.0083), and Az (*P* < 0.0167), a two-sided *P* < 0.05 was considered statistically significant.

## 3. Results

Totally 271 breast masses (from 242 patients) were examined. The maximum diameters of the masses ranged from 6 mm to 30 mm (mean ± SD: 17.44 ± 6.83 mm). According to the maximum diameters, those masses were classified into a small-size group (6–14 mm) and a large-size group (15–30 mm). All masses were proven to be solid histologically after surgery. Histopathologic analysis revealed 153 benign breast lesions and 118 breast carcinomas (Table 1). Therefore, 271 breast masses were categorized into four subgroups: small benign masses (SBM), big benign masses (BBM), small malignant masses (SMM), and big malignant masses (BMMs). The sizes of masses of various subgroups are shown in Table 2.

### 3.1. VTQ

**Figure 2:**
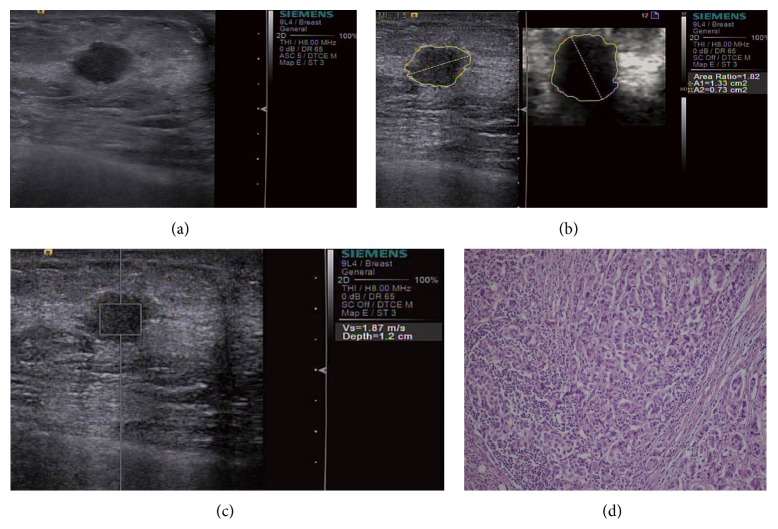
A small invasive ductal carcinoma in a 31-year-old woman. (a) B-mode US image shows a 10 mm hypoechoic, lobular mass. (b) VTI shows Pattern 4 and an AR of 1.82; (c) VTQ measures a SWV of 1.87; (d) histopathologic examination shows that the mass is rich in cancer cells with lymphocyte infiltration and short of fibrous tissue (hematoxylin-eosin, original magnification ×100).

**Figure 3:**
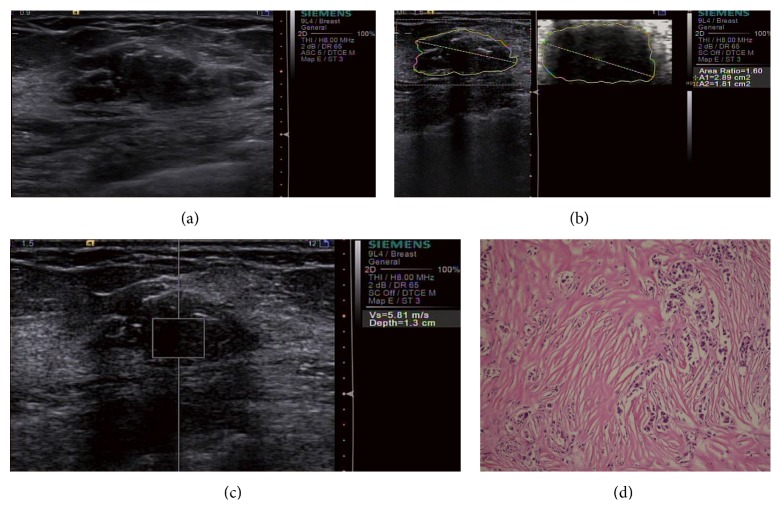
A big invasive ductal carcinoma in a 56-year-old woman. (a) B-mode US image shows a 24 mm irregular hypoechoic mass with ill-defined margins, coarse calcification, and microcalcification; (b) VTI shows Pattern 4 and an AR of 1.60; (c) VTQ measures a SWV of 5.81 m/s; (d) histopathologic examination shows stromal fibrous proliferation with hyaline change and relatively few cancer cells (hematoxylin-eosin, original magnification ×100).

In Table 2, SWVs of masses of each subgroup and comparisons between the groups are shown. SWVs of total malignant masses (TMM) were significantly greater than those of total benign masses (TBM) (*P* < 0.001). In addition, significant differences between SWVs of masses of different subgroups were found (*P* < 0.001): SWVs of SMM and BMM were significantly greater than those of SBM and BBM (*P* < 0.001); also, SWVs of BMM were greater than those of SMM (*P* < 0.001) and SWVs of BBM were greater than those of SBM (*P* < 0.001) (Figures 2 and 3).

The Pearson correlation analysis demonstrated that SWVs of solid breast masses were correlated with mass sizes (*r* = 0.57 for malignant masses, *P* < 0.001; *r* = 0.34 for benign masses, *P* < 0.001).

### 3.2. VTI

Pattern classification of VTI is demonstrated by Table 3. There were significant differences of VTI patterns between TBM and TMM, between SBM and SMM, and between BBM and BMM (*P* < 0.001). However, no differences were identified by comparisons between SBM and BBM (*P* = 0.594) and between SMM and BMM (*P* = 1.000). All Pattern 1 (*n* = 10) and Patten 2 (*n* = 78) masses were benign, whereas most malignant masses (109/118) fell under Pattern 4.

ARs were calculated for 232 masses of Patterns 1, 3, and 4. ARs of masses of each subgroup and comparisons between the groups are shown (Table 2). ARs of TMM were significantly greater than those of TBM (*P* < 0.001). Significant differences of ARs were also found between the subgroups (*P* < 0.001): ARs of SMM and BMM were significantly greater than those of SBM and BBM (*P* < 0.001). However, no significant differences were found between SBM and BBM (*P* = 0.187) and between SMM and BMM (*P* = 0.907) (Figures 2 and 3).

### 3.3. Diagnostic Performance

The cutoff values for SWVs, VTI patterns, and ARs of masses were calculated by receiver operating characteristic curves (ROC). The cutoff values for the SWVs, VTI patterns, and ARs of small masses were 2.545, 4, and 1.155, respectively; those of big masses were 3.575, 4, and 1.035, respectively; and those of total masses were 3.05, 4, and 1.14, respectively. When the presence of those features exceeding the cutoff values was considered a criterion of malignancy, the diagnostic performance of ARFI for solid breast masses of different size groups was evaluated (Table 4).

**Figure 4:**
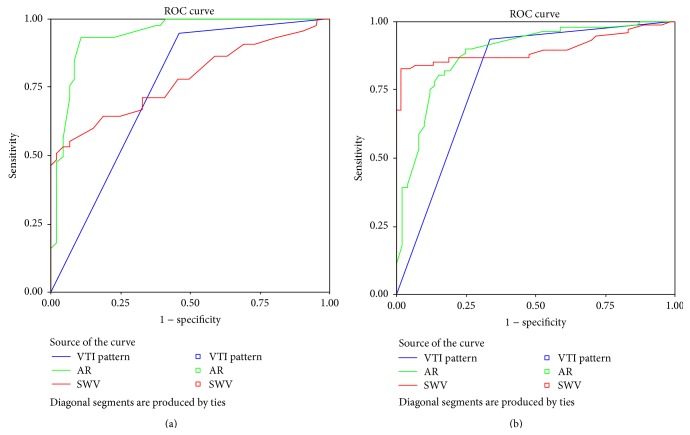
Receiver operating characteristic curves for VTI pattern, AR and SWV for (a) small solid breast masses and (b) big solid breast masses.

For small masses, the Az of AR was significantly larger than either that of SWV (0.942 versus 0.779; *z* = 5.785, *P* < 0.001) or that of VTI pattern (0.942 versus 0.745; *z* = 6.382, *P* < 0.001), while no significant difference was found between the Az of SWV and that of VTI pattern (0.790 versus 0.786; *z* = 0.118, *P* = 0.906) (Figure 4).

For big masses, the Az of VTI pattern was significantly less than either that of SWV (0.838 versus 0.903; *z* = 2.685, *P* = 0.008) or that of AR (0.802 versus 0.886; *z* = 3.088, *P* = 0.002); no significant difference was found between the Az of SWV and that of AR (0.898 versus 0.886; *z* = 0.547, *P* = 0.584) (Figure 4).

It was observed that 44.4% (20/45) of the SMM had SWVs below the 2.545 m/s threshold (Figure 2), whereas 30.8% (4/13) of the ductal carcinomas in situ (DCISs) had SWVs above the threshold. Notably, 84.6% (11/13) of the DCISs fell under Pattern 4, and 76.9% (10/13) of the DCISs had ARs above the threshold. In addition, 100% (3 of 3) of mucinous carcinomas had SWVs below the threshold, all of which fell under Pattern 4 and had ARs above the threshold.

## 4. Discussion

Breast elastography has recently been subject to substantial attention as it has proven to reach an adequate specificity and a high negative predictive value in combination with US [6]. Compared with static elastography, ARFI is a reliable method which provides objective and quantitative elasticity measurements. Several clinical studies have reported that ARFI classification was at least as accurate as US in distinguishing between benign and malignant breast lesions [4–9, 12, 13]. Furthermore, combined with US, ARFI has the potential to improve the accuracy of Breast Imaging Reporting and Data System (BI-RADS) US categorization of breast masses and avoid the need for unnecessary biopsies [4, 8, 13, 14].

In this study, we demonstrated that SWV is a valid feature for differentiating between benign and malignant solid breast masses and a cutoff value of 3.05 m/s for the total mass group. It was additionally found that SWVs of BMM were greater than those of SMM (*P* < 0.001). Likewise, SWVs of BBM were greater than those of SBM (*P* < 0.001). Therefore, it is essential to group solid breast masses according to their sizes when the differential diagnostic value of SWV is being assessed. Otherwise, adopting a 3.05 m/s cutoff value can lead to an increased number of SMM being missed or BBM misdiagnosed.

Our results have also shown that SWV of mass was positively associated with mass size (*r* = 0.57 for malignant masses, *P* < 0.001; *r* = 0.34 for benign masses, *P* < 0.001). Such results may suggest that, with the growth and development of a mass, alterations occur in breast tissue and result in changes of its physical properties, such as stiffness [15]. For benign tumors, increased stiffness can be attributed to fibroplasia, hyaline degeneration, or calcification, while, for malignant ones, intrinsic tumor biologic factors leading to extracellular matrix (ECM) deposition and contraction can account for increasing tissue stiffness [16–18]. Hence, it is reasonable to group solid breast masses according to their size to assess the diagnostic value of SWV.

With 3.575 being the optimal cutoff value for big mass groups, SWV achieved an adequate diagnostic efficiency. However, 44.4% (20/45) of SMM had SWVs of less than the 2.545 m/s cutoff, indicating a low sensitivity (55.6%) of SWV for small mass groups.

In this study, all of Pattern 1 (*n* = 10) and Pattern 2 (*n* = 78) masses were benign, whereas most malignant masses (109/118) fell under Pattern 4. Furthermore, there were also shown significant differences of VTI patterns between malignant and benign breast masses of the same size group (*P* < 0.001). With 4 as the optimal cutoff value, VTI pattern can reach high sensitivities and NPVs for both small and big masses. Especially for small masses, VTI pattern significantly improved the sensitivity (93.3% versus 55.6%), compared with SWV.

AR denotes the discordance of the mass area on the VTI elastogram and on the corresponding B-mode image. Elastography enhances the difference of contrast level between cancerous and noncancerous tissues [19] and potentially provides a better visualization of the mass margins than US is able to. A known mechanism relates to a desmoplastic reaction occurring in many malignant breast tumors [20, 21], which results in infiltrating surrounding tissue and makes a malignant mass appear larger by elastography than by US. Several studies proposed that an elastogram/B-mode ratio ≥1 is suggestive of malignancy [14, 19]. In this study, ARs were calculated for masses of Patterns 1, 3, and 4. ARs of malignant mass groups were greater than those of benign mass groups (*P* < 0.001), in agreement with the former studies. Using cutoff values of 1.155, 1.035, and 1.14 for small, big, and total masses, respectively, AR achieved good diagnostic efficiencies. For small masses, the diagnostic efficiency of AR was significantly better than that of SWV (0.942 versus 0.779; *z* = 5.785, *P* < 0.001), while, for big masses, it is similar to that of SWV (0.886 versus 0.898; *z* = 0.547, *P* = 0.584). These results have highlighted that AR is a good feature for differentiating between benign and malignant solid breast masses.

In this study, 30.8% (4/13) of DCISs had SWVs above the threshold, while 84.6% (11/13) of DCISs fell under Pattern 4 and 76.9% (10/13) had ARs above the threshold, suggesting that VTI is more sensitive than VTQ for detecting DCIS. Furthermore, when mucinous carcinoma, a kind of softer breast cancer because of the abundant mucus it contains [22], is concerned, our results showed that 100% (3/3) of them with SWVs below the threshold could not be differentiated from benign disease by VTQ. Notwithstanding, all of them fell under Pattern 4 and had ARs above the threshold; hence, they could be correctly diagnosed as malignancy. These results indicate that, for detection of this specific type of malignancy, VTI has better diagnostic efficiency than VTQ.

This study has several limitations. First, the dimension of VTQ ROI is fixed and that of VTI ROI is restricted, which would limit clinical utility. Second, only a relatively small number of malignant cases and types have been examined. Third, ARs were unavailable for masses of Pattern 2, resulting in missing data. Fourth, the association between stiffness and histological grade has not been investigated.

In conclusion, ARFI is helpful for differential diagnosis of benign and malignant solid breast masses. For big masses, SWV and AR are better measures than VTI pattern and seem to have similar diagnostic potential. Nevertheless, as small masses tend to have a low stiffness measured by SWV, VTI should be preferred, and AR can be considered the best feature.

## Figures and Tables

**Table 1:** Histological diagnosis of 271 solid breast masses.

Pathologic diagnosis	Number of cases		
	Small	Big	Total
Malignant masses			
Invasive ductal carcinoma	33	64	97
Ductal carcinomas in situ	8	5	13
Mucinous carcinoma	2	1	3
Invasive lobular carcinoma	1	1	2
Neuroendocrine carcinoma	0	1	1
Invasive papillary carcinoma	0	1	1
Lymphoma	1	0	1
Benign masses			
Fibroadenoma	48	73	121
Adenosis	11	7	18
Intraductal papilloma	7	4	11
Benign phyllodes tumor	0	2	2
Hamartoma	0	1	1

**Table 2:** Size, ARs, and SWVs of masses of multiple groups and their comparisons.

Feature	SBM *n* = 66	BBM *n* = 87	TBM *n* = 153	SMM *n* = 45	BMM *n* = 73	TMM *n* = 118
Size, mm	10.56 ± 2.39	21.33 ± 4.94	16.68 ± 6.71	11.64 ± 2.08	22.60 ± 5.29	18.42 ± 6.89
SWV, m/s	1.82 ± 0.45^g^	2.18 ± 0.60	2.03 ± 0.57	3.66 ± 2.35^b,c^	6.74 ± 2.77^d,e,f^	5.57 ± 3.01^a^
AR	0.92 ± 0.20^h^	0.97 ± 0.21^i^	0.95 ± 0.21	1.46 ± 0.34^k,l^	1.43 ± 0.34^m,n^	1.44 ± 0.38^j^

Note: data are mean ± SD.

^a^ *P* < 0.001, compared with TBM.

^b,c^ *P* < 0.001, compared with SBM and BBM, respectively.

^d,e,f^ *P* < 0.001, compared with SBM, BBM, and SMM, respectively.

^g^ *P* < 0.001, compared with BBM.

^h^For SBM, *n* = 50, because ARs were unavailable in 16 masses of Pattern 2.

^i^For BBM, *n* = 64, because ARs were unavailable in 23 masses of Pattern 2.

^j^ *P* < 0.001, compared with TBM.

^k,l^ *P* < 0.001, compared with SBM and BBM, respectively.

^m,n^ *P* < 0.001, compared with SBM and BBM, respectively.

**Table 3:** VTI patterns in 271 solid breast masses.

VTI pattern	SBM (*n* = 66)	BBM (*n* = 87)	TBM (*n* = 153)	SMM (*n* = 45)	BMM (*n* = 73)	TMM (*n* = 118)
Pattern 1	2	3	5	0	0	0
Pattern 2	16	23	39	0	0	0
Pattern 3	29	44	73	3	6	9
Pattern 4	19	17	36	42	67	109

**Table 4:** Diagnostic performance of ARFI for breast masses from different size groups.

Group and feature	Sensitivity (%)	Specificity (%)	PPV (%)	NPV (%)	Accuracy (%)	Az (95% CI)
Small (*n* = 111)						
SWV ≥ 2.545	55.6 (25/45)	95.5 (63/66)	89.3 (25/28)	75.9 (63/83)	79.3 (88/111)	0.790 (0.743–0.837)
VTI pattern = 4	93.3 (42/45)	71.2 (47/66)	68.9 (42/61)	94.0 (47/50)	80.2 (89/111)	0.786 (0.739–0.833)
AR ≥ 1.155^a^	93.3 (42/45)	90 (45/50)	89.4 (42/47)	93.8 (45/48)	91.6 (87/95)	0.942 (0.916–0.967)
Big (*n* = 160)						
SWV ≥ 3.575	82.2 (60/73)	98.9 (86/87)	98.4 (60/61)	86.9 (86/99)	91.3 (146/160)	0.903 (0.874–0.931)
VTI pattern = 4	91.8 (67/73)	80.5 (70/87)	79.8 (67/84)	92.1 (70/76)	85.6 (137/160)	0.838 (0.802–0.875)
AR ≥ 1.035^b^	89.0 (65/73)	79.7 (51/64)	83.3 (65/78)	86.4 (51/59)	84.7 (116/137)	0.886 (0.856–0.917)
Total (*n* = 271)						
SWV ≥ 3.05	69.5 (82/118)	97.4 (149/153)	95.3 (82/86)	80.5 (149/185)	85.2 (231/271)	0.846 (0.820–0.873)
VTI pattern = 4	92.4 (109/118)	76.5 (117/153)	75.1 (109/145)	92.9 (117/126)	83.4 (226/271)	0.815 (0.786–0.844)
AR ≥ 1.14^c^	85.6 (101/118)	87.7 (100/114)	87.8 (101/115)	85.5 (100/117)	86.6 (201/232)	0.909 (0.889–0.930)

Note: ^a^ARs were calculated for masses of Patterns 1, 3, and 4, *n* = 95; ^b^ARs were calculated for masses of Patterns 1, 3, and 4, *n* = 137; ^c^ARs were calculated for masses of Patterns 1, 3, and 4, *n* = 232.
